# Utility of radioguided surgery in the intraoperative localization of neuroendocrine tumors: Report of 3 cases

**DOI:** 10.22038/aojnmb.2025.87066.1623

**Published:** 2026

**Authors:** Jon A. Uña-Gorospe, Sonia Romero-Acevedo, Laura Mora-Martín

**Affiliations:** 1 Nuclear Medicine Department, Hospital Universitario Nuestra Señora de Candelaria, Santa Cruz de Tenerife, Spain; 2 Nuclear Medicine Department. Hospital Universitario de Canarias, Carretera, Santa Cruz de Tenerife, Spain; 3 Endocrinology Department. Hospital Universitario Nuestra Señora de Candelaria, Santa Cruz de Tenerife, Spain

**Keywords:** Gamma probe, Tektrotyd, Neuroendocrine, ROLL, GOSTT A B S T R A C T

## Abstract

Neuroendocrine tumors (NETs) represent a heterogeneous group of neoplasms originating from neuroendocrine cells. Overexpression of somatostatin receptors is typically correlated with tumor differentiation.

Localization and characterization of the primary tumor are essential, as radical surgery remains the treatment of choice for resectable disease. Additionally, identifying metastatic disease and assessing its extent are crucial for disease staging and monitoring response aimed at reducing total tumor volume.

Radioguided surgery based on the ability to detect somatostatin receptor overexpression, is a valuable tool that aids in the identification of microscopic and occult endocrine tumors.

In this context, we present three patients who achieved improved outcomes due to enhanced detection and identification of previously indeterminate or undetected lesions on prior imaging or even the detection of tumors that were not easily visualized without radioguided assistance.

Complete tumor removal is a key prognostic factor in patients with NETs, improving quality of life and reducing the risk of tumor recurrence or locorregional metastasis. Achieving R0 or R1 resections has been associated with better survival outcomes.

The successful implementation of radioguided surgery for NETs requires a multidisciplinary approach, both surgeons and nuclear medicine specialists playing a relevant role, and who must be aware of its prognostic significance.

## Introduction

 Neuroendocrine tumors (NETs) represent a heterogeneous group of neoplasms that originate from neuroendocrine cells, derived from the ectodermal cells of the neural crest, endocrine glands, islets, and the diffuse neuroendocrine system. They are characterized by producing neuropeptides such as synaptophysin or chromogranin A, and by hormone secretion depending on the organ of origin, leading to great clinical variability.

 All NETs are considered potentially malignant, with prognosis influenced by symptoms, site, histological type, and grade of differentiation (mitotic index and Ki-67), and even their metastatic potential varies widely. 

 Overexpression of somatostatin receptors (SSTR) generally correlates with tumor differentiation, distinguishing well-differentiated NETs (grades G1–G3) from poorly differentiated neuroendocrine carcinomas.

 Precise localization of the primary tumor is essential, as radical surgery remains the treatment of choice for resectable disease. Accurate identification of metastatic spread is crucial for disease staging and monitoring tumor volumen reduction therapies, since occult primary NETs increase the risk of dissemination and can lead to high rates of postoperative recurrence.

 Radio-guided surgery using different tracers has emerged as a valuable tool for intraoperative localization of NET lesions (1). Several approaches have been described, including sentinel lymph node mapping, radioguided occult lesion localization (ROLL), or Guided intraOperative Scintigraphic Tumor Targeting (GOSTT), each with specific methodological nuances depending on the surgical indication.

 Here we present three clinical cases in which radioguided surgery provided clear benefits in the intraoperative management of NETs.

***Case 1***

 A 62-year-old man was incidentally found to have a gastric lesion on CT in 2014. Biopsy confirmed a WHO 2012 G2 NET (Ki-67: 5.2%). The initial ^111^In-Octreoscan® was negative. The patient underwent endoscopic gastric resection with follow-up until 2021, when ^99m^Tc-Tektrotyd® (^99m^Tc-HYNIC TOC) scintigraphy revealed a 1 cm lesion adjacent to the duodenal loop, suggestive of a pathological lymph node. 

 After a 370 MBq of ^99m^Tc-Tektrotyd® injection, radioguided surgery was performed. Intraoperative gamma probe survey identified an additional focal activity in the duodenal wall, corresponding to a 1 cm nodule beneath the serosa. Mean counts were approximately 60 cps for both nodules, in contrast with background levels of 17–20 cps in the intestine and 50 cps in the liver. Resection beds for both lesions were re-checked with the probe, confirming significant reduction in activity.

 Follow-up for G1 NET (T2N1) demonstrated persistent but gradually decreasing levels of CgA and gastrin, without any evidence of recurrence to date.

***Case 2***

**Figure 1:**
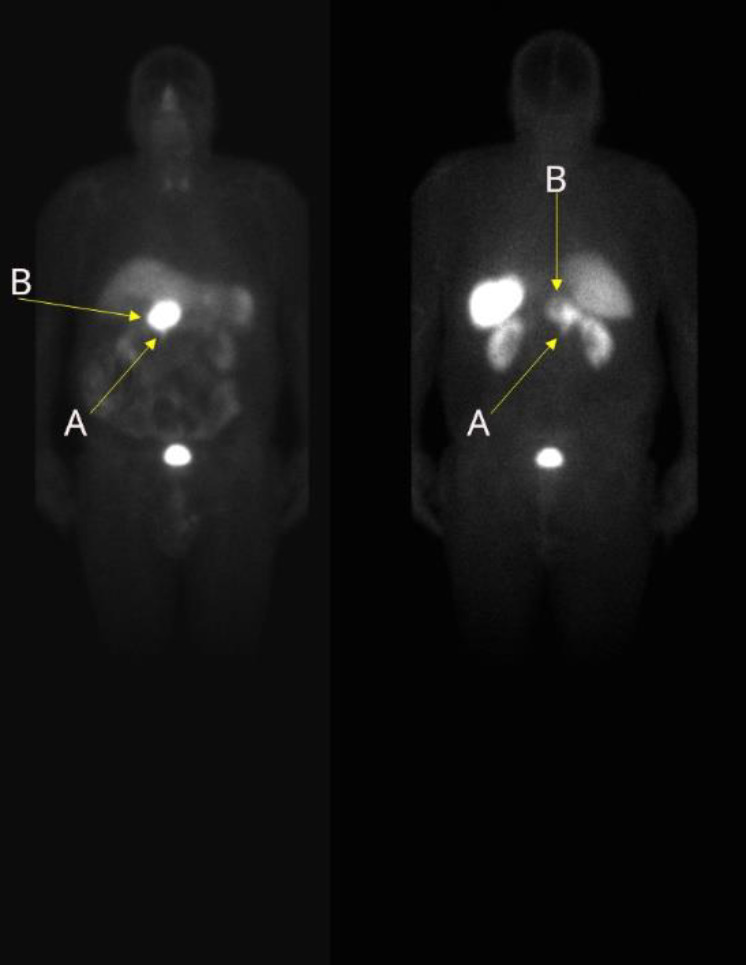
Planar ^99m^Tc-Tektrotyd® scintigraphy of Case 2, showing two lesions with high SSTR expression: (**A**) duodenal primary tumor, (**B**) peripancreatic lymph node metastasis

 A 77-year-old patient presented with a well-differentiated duodenal NET measuring 15 mm and a 4 cm peripancreatic lymph node metastasis, with elevated chromogranin A levels of 1163 ng/ml. A ^99m^Tc-Tektrotyd® scintigraphy with 740 MBq, performed with planar and SPECT-CT acquisition, showed a high SSTR expression consistent with Krenning grade 4 in both lesions, and revealed additional suspicious uptake near the hepatic hilum (Figure 1).

**Figure 2:**
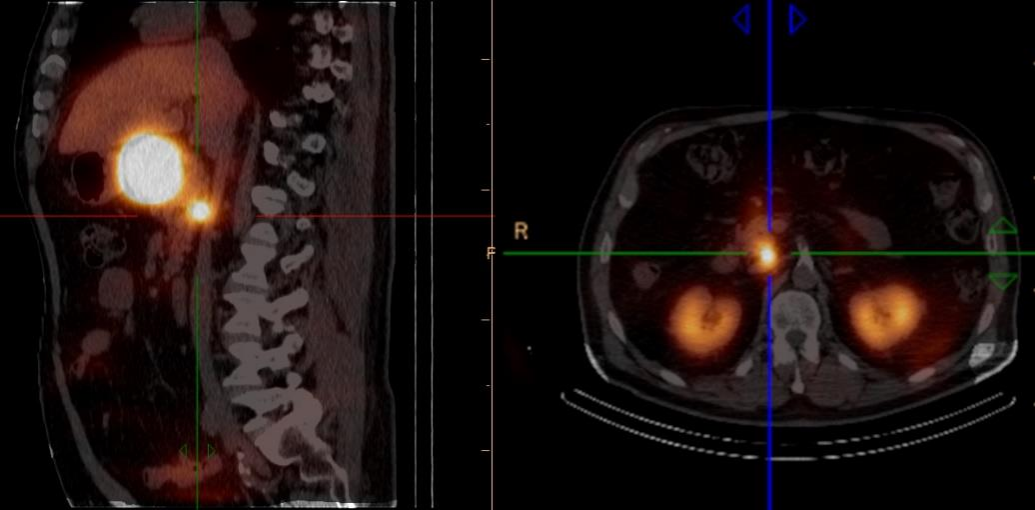
SPECT-CT of Case 2 after 740 MBq ^99m^Tc-Tektrotyd®. Transaxial and sagittal fused images demonstrate intense uptake in both the enlarged adenopathy and the primary duodenal lesion

 Based on these findings, radioguided surgery was then performed after a 400 MBq ^99m^Tc-Tektrotyd injection. Intraoperative a gamma probe was used to asses background counts on the normal liver (900 cps) and the digestive tract (800 cps), whereas the primary duodenal lesion, the metastatic node and a retroportal hiliar node rose to 2000 cps, 4500 cps and 1800 cps, respectively. Notably, the hiliar node showed twice the normal liver background uptake, although it had only appeared faintly on SPECT-CT, and was not visible on planar imaging (Figure 2).

***Case 3***

 A male liver transplant recipient developed a focal lesion in the graft, suspicious for NET metastasis, based on positive ^99m^Tc-Tektrotyd® imaging findings with unknown primary tumor. Serial ^99m^Tc-Tektrotyd® scintigraphy later revealed a focal radiotracer deposit in the wall of an intestinal loop in the left flank. Radioguided surgery was performed following administration of 370 MBq of ^99m^Tc-Tektrotyd®, to identify the intestinal lesion and to investigate possible lymph node foci not detected by the gamma camera or in previous CT.

**Figure 3:**
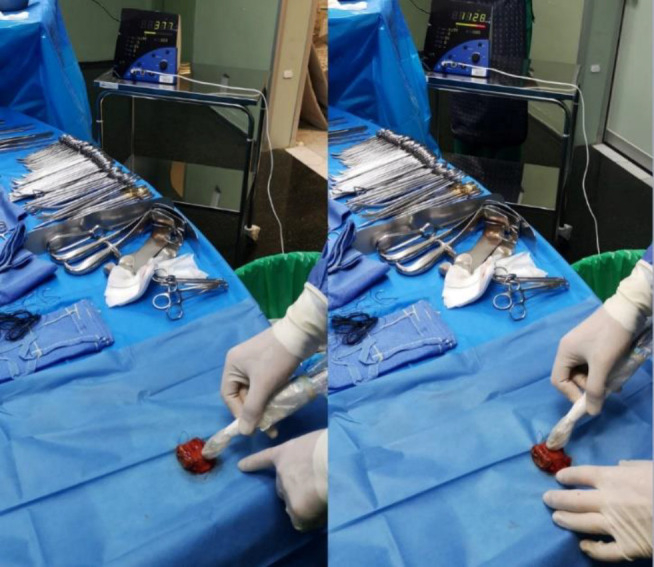
Intraoperative photographs of Case 3 showing gamma probe use during jejunal lesion resection

 During surgery, a jejunal lesion was identified, showing high counts of 1325 cps, relative to background intestinal activity of 250 cps. No other foci were found. Pathology results confirmed a well-differentiated NET G2 with R0 resection(Figure 3).

.

## Discussion

 The clinical usefulness of radioguided surgical techniques with radiotracers has been demonstrated for several decades. The first description of a scintigraphic surgical probe dates back to 1981, when Harvey et al. reported its technical and clinical characteristics in bone lesions (2). Since then, gamma probes have evolved and demonstrated their utility at identifing and localizing several lesions, while minimizing post-surgical side effects, extending their application to parathyroid surgery (3), sentinel lymph node detection in breast cancer (4), and radioguided occult lesion localization (ROLL) (5).

 Although less frequently reported, radioguided surgery has also been applied to neuroendocrine tumors (NETs) since the 1990s (6,7) and to tumors derived from the neural crest (8). 

 At our center, gamma probe-guided procedures are routinely used for sentinel lymph node mapping for breast, melanoma, and head and neck tumors, as well as to help at parathyroid detection, and occult lesions after intralesional injection of ^99m^Tc-MAA. More occasional uses include ^123^I-MIBG in neural crest tumors (8,9) and somatostatin analogues labeled with ^99m^Tc or ^68^Ga for NETs (7, 10-12).

 Surgical resection remains the most effective curative option for early-stage NETs, and achieving R0 resection is a major prognostic factor, associated with improved survival outcomes and reduced risk of metastasis (13). 

 Radioguided surgery can contribute to this goal by reducing operative time, minimizing surgical manipulation, and increasing precision. Adams et al. reported that radioguided surgery is among the most sensitive intraoperative tools for detecting microscopic and occult endocrine tumors (14). Our cases illustrate this added value: identifying a duodenal primary missed by conventional imaging, confirming suspicious nodes not clearly visualized in scintigraphy, and localizing a jejunal lesion in a transplant recipient.

^68^Ga/^18^F-DOTA PET-CT is now considered the gold standard for NET imaging because of its higher sensitivity, superior spatial resolution, and stronger affinity for SSTR compared with SPECT. While PET-CT often detects lesions missed on SPECT, it does not replace the role of intraoperative guidance. PET provides excellent preoperative staging, but lacks real-time applicability during surgery. The gamma probe allows detection and verification of lesions intraoperatively, including subcentimetric or low-uptake foci that may remain indeterminate even on PET. It also helps confirm complete removal of disease, guiding the surgeon toward R0 resection and reducing the need for extensive exploration. Thus, PET-CT and radioguided surgery should be regarded as complementary techniques: PET optimizes preoperative planning, while radioguided surgery enhances intraoperative precision and outcomes.

 Radioguided surgery for NETs therefore represents a multidisciplinary approach that relies on close collaboration between nuclear medicine physicians and surgeons. Proper teamwork facilitates accurate localization, reduces surgical time and morbidity, and improves patient outcomes.

## Conclusion

Radioguided surgery using ^99m^Tc-HYNIC-TOC provides a valuable intraoperative tool for localizing neuroendocrine tumors with SSTR overexpression. Although still underreported, our cases illustrate its ability to confirm indeterminate lesions, detect subcentimetric foci, and support complete resection. 

 While ^68^Ga/^18^F-DOTA PET-CT remains the gold standard for preoperative NET imaging, radioguided surgery complements it by offering real-time guidance during surgery, reducing surgical time and morbidity, and increasing the likelihood of achieving R0 resection. This approach underscores the importance of close collaboration between nuclear medicine specialists and surgeons in the management of NETs.
